# Positive selection on rare variants of *IGF1R* and *BRD4* underlying the cold adaptation of wild boar

**DOI:** 10.1186/s12711-025-00986-y

**Published:** 2025-07-16

**Authors:** Jianhai Chen, Ivan Jakovlić, Mikhail Sablin, Shengqian Xia, Zhixiang Xu, Yapin Guo, Renzuo Kuang, Jie Zhong, Yangying Jia, Nhien Thuy Thi Tran, Hao Yang, Hong Ma, Nikica Šprem, Jianlin Han, Di Liu, Yunxia Zhao, Shuhong Zhao

**Affiliations:** 1 Key Laboratory of Agricultural Animal Genetics, Breeding and Reproduction, Ministry of Education, College of Animal Science and Veterinary Medicine, Huazhong Agricultural University, Wuhan, 430070 China; 2 Department of Ecology and Evolution, The University of Chicago, 1101E 57th Street, Chicago, IL 60637 USA; 3 Institutes for Systems Genetics, Frontiers Science Center for Disease-Related Molecular Network, West China Hospital, Sichuan University, Chengdu, 610041 Sichuan China; 4 State Key Laboratory of Grassland Agro-Ecosystems, and College of Ecology, Lanzhou University, Lanzhou, 730000 China; 5 Bio-Transduction Lab, Wuhan, China; 6 Zoological Institute Russian Academy of Science, Universitetskaya Nab. 1, Saint Petersburg, 199034 Russia; 7 Institute of Rare Diseases, West China Hospital of Sichuan University, Sichuan University, Chengdu, 610000 China; 8 National Institute of Animal Sciences, Hanoi, Vietnam; 9 Key Lab of Agricultural Animal Genetics, Breeding and Reproduction, Ministry of Education, College of Animal Science and Technology, Huazhong Agricultural University, Wuhan, 430070 China; 10 West China-Washington Mitochondria and Metabolism Research Center, Key Lab of Transplant Engineering and Immunology, MOH, Regenerative Medicine Research Center, West China Hospital, Sichuan University, No. 88, Keyuan South Road, Hi-Tech Zone, Chengdu, 610041 China; 11 Heilongjiang Academy of Agricultural Sciences, Haerbin, China; 12 Department of Fisheries, Apiculture, Wildlife Management and Special Zoology, Faculty of Agriculture, University of Zagreb, Zagreb, Croatia; 13 Yazhouwan National Laboratory, Sanya, 572024 Hainan China

## Abstract

**Background:**

Domestic piglets often die of hypothermia, whereas Eurasian wild boar (Sus scrofa) thrives from tropical lowlands to subarctic forests. The thermoregulation of wild boar offers a natural experiment to uncover the genetic basis of cold adaptation.

**Methods:**

We conducted whole-genome resequencing on wild populations from cold regions (northern and northeastern Asia, with six samples) and warm regions (southeastern Asia and southern China, with five samples). By integrating publicly available data, we compiled a core dataset of 48 wild boar samples and an extended dataset of 445 wild boar and domestic pig samples to identify candidate genes related to cold adaptation. To investigate the functional effects of two candidate variants under positive selection, we performed CUT&Tag and RNA-seq using the northeastern Asian Min pig breed as a proxy for a cold-adapted population.

**Results:**

Our study identified candidate genes associated with cold adaptation, which are significantly enriched in thermogenesis, fat cell development, and adipose tissue pathways. We discovered two enhancer variants under positive selection: an intronic variant of *IGF1R* (rs341219502) and an exonic variant of *BRD4* (rs327139795). These variants exhibited the highest differentiation between populations of wild boar and domestic pigs in cold and warm region populations. Furthermore, these rare variants were absent in outgroup species and warm-region wild boars but were nearly fixed in cold-region populations. The H3K27ac CUT&Tag profiling revealed that the rs341219502 variant of *IGF1R* is linked to the gain of novel binding sites for three transcription factors involving regulatory changes in enhancer function. In contrast, the rs327139795 variant of *BRD4* may result in the loss of a phosphorylation site due to an alteration in the amino acid sequence.

**Conclusion:**

Our study identified candidate genes for cold adaptation in wild boar. The variant rs341219502 in the *IGF1R* enhancer and the variant rs327139795 in the *BRD4* exon, both of which were under positive selection and nearly fixed in populations from cold regions, suggest they may have originated de novo in these populations. Further analysis indicated that rs341219502 could influence enhancer function, while rs327139795 may affect amino acid alterations. Overall, our study highlights the adaptive evolution of genomic molecules that contribute to the remarkable environmental flexibility of wild boar.

**Supplementary Information:**

The online version contains supplementary material available at 10.1186/s12711-025-00986-y.

## Background

One of the most fundamental questions in evolution is to understand how populations adapt to new environments [1, 2]. Peripheral or marginal populations, which inhabit the edge of a species' distribution area, provide a valuable model for exploring this question. Peripheral populations often migrate from their ancestral territories to adapt to exploit new niches through population expansion. However, they face harsher and sometimes insurmountable environmental conditions at these boundary zones, such as extremely low temperatures and a scarcity of food resources, which can hinder their further expansion. The challenges presented by these survival conditions make peripheral populations ideal natural experiments for investigating the genetic bases of novel adaptive strategies in response to environmental constraints.

Advancements in sequencing technology and population genetics are increasingly allowing for the identification of functional genetic variants under natural selection, enabling organisms to adapt to new environments [3–7]. Strong selective forces in derived populations can leave distinct genomic signals that differentiate them from source populations [2, 8]. Research indicates that 1 to 15% of genes in the mammalian genome may experience positive Darwinian selection [9–11]. For example, in humans, it is estimated that approximately 10% of genes are under positive selection, although the concordance rates among different statistical tests typically range from 8 to 27% [10, 11].

To identify selective signals in peripheral populations, studies often examine patterns across multiple genomic parameters, including allele frequencies [2, 12, 13], nucleotide diversity [14–16], and haplotype segregation [17, 18]. Human populations have demonstrated adaptability to various environments, from tropical regions in Africa to the cold peripheral regions of Siberia. A genome-wide scan analysing haplotype and allele frequency patterns in Siberian populations revealed selective signals associated with genes that contribute to cold adaptation [3]. One example is the identification of a globally low-frequency nonsynonymous variant in the *CPT1A* gene, which is thought to be the most likely causative mutation, as it has a high allele frequency in local Siberian populations [19].

In this study, we focus on the wild boar (*Sus scrofa*), a species known for its remarkable adaptability and wide distribution across various climatic regions in Eurasia. Wild boar occupy diverse ecological niches, ranging from the humid tropics of Southeast Asia, through temperate zones, to the extreme environments of the Qinghai-Tibet plateau and subarctic Siberia [20, 21].

Phylogenetic analyses indicate that the Eurasian wild boar diverged from a clade of closely related *Sus* species at the beginning of the Pliocene epoch, approximately 5.3 to 3.5 million years ago, in tropical Asia [22]. Between 1 and 2 million years ago, the species expanded beyond tropical Asia, establishing several geographically distinct populations across Eurasia [20]. Biogeographic studies have traced the migratory path of wild boars from southern to northern Asia [23, 24].

This long-range migration of wild boar suggests that the populations in tropical regions acted as source populations, while those in Siberian areas are peripheral populations. By examining genomic changes in these peripheral wild boar populations, we can investigate the genetic basis of traits that are subject to positive selection in response to novel climatic challenges under cold environments.

Wild boar has migrated from tropical Asia and established their northernmost natural habitat in Siberia, reaching latitudes as far north as 61°N [21]. Given their tropical origins, we hypothesise that the genomes of wild boar populations in cold regions may show signs of adaptation to these environments. However, this hypothesis has not yet been empirically tested [21].

In our study, we conducted whole-genome sequencing of wild boar populations from both tropical Asian regions (specifically Vietnam, with five samples) and cold Asian regions (Siberia, with six samples). We also included 445 publicly available samples from both domestic pigs and wild boars for comparison [17, 25–34].

Our research focused on identifying candidate genes and associated biological pathways that may underlie the cold adaptation of wild boar. We examined whether these candidate genes have been highlighted in previous studies involving other species. By analysing these selectively advantageous genes, we discovered the leading genetic variants that exhibit significant differentiation between warm- and cold-tolerant populations, as well as site-level signals indicating selective sweeps among all regulatory and missense variants.

Additionally, we investigated the functional implications of these leading variants through experiments, including the Cleavage Under Targets and Tagmentation (CUT&Tag) and RNA-seq. Our study provides valuable insights into the positively selected genes and rare variants that may contribute to the bioclimatic adaptation of wild boar in cold regions.

## Methods

### Whole genome sequencing and variants calling

Genomic DNA was extracted from hair follicles of five Vietnamese wild boars (Son La province, Vietnam, ~ 20°N), three wild boar from the Novosibirsk region (Novomyhaylovka village, Kochenyovskiy district, Latitude 55°17′35ʺN, Longitude 81°48′38ʺE), one wild boar from Tyva (~ 51°N), one wild boar from Buryat (~ 51°N), and one wild boar from Zabaykalsky Krai (~ 52°N). The extraction protocol was based on a modified phenol–chloroform method [35]. The procedure is summarised as follows: more than ten hair follicles were collected from each pig and transferred into a 1.5 mL microcentrifuge tube. A lysis buffer containing SDS and Proteinase K was added, and the samples were incubated at 56 °C for 1–2 h to ensure complete tissue digestion. After lysis, an equal volume of phenol/chloroform/isoamyl alcohol (25:24:1) was added and mixed thoroughly. Following centrifugation, the clear aqueous phase was transferred to a new tube, and genomic DNA was precipitated using two volumes of pre-chilled ethanol. Ethanol was chosen instead of isopropanol to avoid interference in downstream applications due to the lower volatility of isopropanol. The DNA was pelleted through high-speed centrifugation, washed, and resuspended in TE buffer. The extracted DNA exhibited A260/A280 ratios between 1.85 and 2.00, with yielded exceeding 4 μg per sample, which was sufficient for downstream PCR-based applications. Whole-genome sequencing (WGS) was performed on all samples using the DNBSEQ-T7 platform (MGI) with a paired-end library (2 × 125 bp).

Whole-genome mapping and calling processes were primarily carried out following established methodology [17, 36, 37]. In brief, whole-genome short-read data for samples representing wild boars, domestic pigs, and outgroup species were cleaned using the fastp software with its default parameters [38]. The cleaned data were then mapped to the genomic reference Sscrofa 11.1 using BWA v0.7.17 [39]. Notably, sex chromosome variants were excluded from further analyses. Despite the availability of several reference-level assembled genomes for pigs, including various domestic breeds [40, 41] and the wild boar [37], we selected Sscrofa11.1 as the reference due to its superior annotation and sequencing quality [42].

For variant calling, we utilised two software programs SAMtools v1.15.1 [43] and GATK v4.2.6.1 (https://gatk.broadinstitute.org/hc/en-us). The primary steps included marking duplicates, recalibrating base quality scores, performing per-sample calling with HaplotypeCaller, and conducting joint-calling with GenotypeGVCFs. We filtered variants using the expression “QUAL < 100.0 || QD < 2.0 || MQ < 40.0 || FS > 200.0 || SOR > 10.0 || MQRankSum < − 12.5 || ReadPosRankSum < − 8.0”. These procedures were applied to both newly sequenced samples and publicly available ones [17, 25–34]. To minimise the impact of samples with low sequencing depth, a minor allele frequency (MAF) threshold of 0.05 and a missing genotyping rate of 0.2 were implemented. Population-based phasing was conducted using Beagle5.2 [44].

### Use of other datasets and grouping strategy

To reduce computational burden while gaining a comprehensive understanding, we designed two datasets: a core dataset focusing on wild boar and an extended dataset that included both wild boar and domestic pigs. The simplified core dataset consisted of 48 wild boar samples and 15 samples from outgroup species (see Additional file 1, Table S1). This core dataset was utilised for nearly all analyses, except for the initial confirmation of population identity and the analysis of allele frequency distributions.

For the core dataset, we included 24 wild boars from northern and northeastern China to represent the cold region population, and 24 wild boars from southern China, Sumatra, and southern Europe (Italy and Greece) to represent the warm region populations. The 15 samples of outgroup species comprised four different *Sus* species: *Sus verrucosus, Sus celebensis, Sus cebifrons,* and *Sus barbatus* [22] (see Additional file 1 Table S1).

Incorporating European pigs into our dataset enabled us to distinguish between alleles of European origin and those that arose locally in Asia, which helped minimise the risk of misinterpreting recent gene flow as indicators of natural selection for cold adaptation. Additionally, while southern Europe differs from tropical Asia in environmental conditions, both regions share a more temperate climate and are closer in latitude compared to Siberia, which is classified as the cold region in this study. For simplicity, we categorise Italy and tropical Asia together under the non-cold region label, referring to them as the warm region in this analysis.

The second dataset was significantly larger, comprising 488 samples that included domestic pigs, wild boar, and outgroup species. This dataset was utilised to confirm the population identity of new samples and to evaluate the distribution of allele frequencies across different geographical populations (see Additional file 1, Table S2). Specifically, the dataset included 11 new samples along with 477 samples from the public databases, consisting of wild boar and domestic pig samples (see Additional file 1, Table S2).

The geographic populations were categorized as follows: European wild boar (EUW, 47 samples), East Asian northern wild boar (EANW, 30 samples), East Asian southern wild boar (EASW, 18 samples), southeastern Asian wild boar (SEAW, 8 samples), and outgroups species (OG, including African warthog, African bush hog, African red river hog, pygmy hog, Southeast Asian *Sus* species, 32 samples).

Additionally, domestic pig samples were classified into the following categories: European domestic pigs (EUD, 186 samples), East Asian northern domestic pigs (EAND, 84 samples), East Asian southern domestic pigs (EASD, 31 samples), and East Asian western domestic pigs (EAWD, which are also Tibetan pigs, 52 samples). After applying filters for MAF (0.05) and genotype quality (0.2), we retained 21,845,142 variants for the extended dataset and 7,804,721 variants for the core dataset (excluding outgroup samples from both) (Table 1).

**Table 1:** Sample size and data sources for whole-genome resequencing

	Wild boar and domestic pigs (WB and DP)		Outgroup species
	New data (WB)	Downloaded data (WB, DP)	Samples
Core dataset	11	37	15
Extended dataset	11	445	32

### Population relationships, ancestry, and gene flow

To investigate whether sample size affects population relationship, we analysed two datasets: a core dataset consisting of 63 genomes of wild boar and outgroup species samples and an extended dataset comprising 488 genomes of wild boar, domestic pigs, and outgroup samples. We began by evaluating and comparing the phylogenetic topologies of the genomes using a distance matrix generated based on identity by state (IBS) (PLINK (v1.9) “-maf 0.05 –geno 0.2 –distance square 1-ibs -thin-count 100,000“).

For the core dataset, we also conducted principal component analysis (PCA) to analyse inter-group relationships and population clustering. To assess potential gene flow between western and eastern Siberian populations, we employed TreeMix analysis [45]. We estimated the optimal number of migration events (m) using OptM inference, applying a model threshold of 99% [46]. We also performed ancestry estimation and admixture analyses using ADMIXTURE v1.3 [47].

### Genome-wide scan for natural selection signals in the North Asian wild boar populations

We conducted a genome-wide scan for signals of natural selection in the North Asian wild boar population based on the core dataset (MAF 0.05) (see Additional file 1 Table S1), using the following four complementary statistics: the fixation index (*F*_*st*_) and nucleotide diversity ratio (θ_π_warm_/θ_π_cold_); the XP-CLR test and nucleotide diversity ratio (θ_π_warm_/θ_π_cold_) [48]; the extended haplotype homozygosity (EHH)-based statistic (ihh12) [49, 50]; and The Hudson–Kreitman–Aguadé (HKA)-like test [51, 52]. To focus on selection analyses, we removed samples of putative European origin based on PCA, ADMIXTURE results, and phylogenetic data. This allowed us to retain closely related samples of Asian origin, representing populations from cold and temperate regions, with a total of 15 samples from cold regions and 21 samples from temperate regions.

The fixation index (*F*_*st*_) and nucleotide diversity (π) are classical parameters used to understand genetic differentiation. It is expected that recent positive selection reduces nucleotide diversity while increasing *F*_*st*_ between two populations that exhibit differentiated phenotypes. Therefore, we anticipated that the selected chromosomal regions in North Asian wild boars will show higher *F*_*st*_ values but lower π values.

The XP-CLR test, a cross-population composite likelihood ratio test based on the site frequency spectrum (SFS), is a powerful test for identifying genomic regions under positive selection within a single population [48, 53]. This test detects such regions by identifying shifts in allele frequencies and patterns of linkage disequilibrium, making it effective for pinpointing both recent and ancient selection events [48, 53].

The method known as ihh12, which is based on haplotype analysis, was developed to detect both hard and soft selective sweeps. It provides greater power than other tools, such as iHS, for identifying soft sweeps [49, 50]. Hard sweeps occur when a beneficial mutation arises and quickly reaches fixation, leading to a reduction of genetic variation in the surrounding region. In contrast, soft sweeps involve multiple beneficial mutations at the same locus or rely on pre-existing genetic variation, resulting in a more gradual and partial reduction in genetic diversity [49, 50].

Additionally, the HKA-like test is grounded in the neutral theory prediction that species divergence (fixed site differences) should correlate with population polymorphism [51]. If selection operates at the population level, we expect a faster reduction in polymorphisms compared to species divergence [52, 54].

These methods and principles have also been extensively applied in medical genetics to identify disease variants [55–57]. In our analysis, we used inter-species differences with numbers of fixed sites (> 60 SNPs) as a measure of divergence between *S. scrofa* and other four *Sus* species (*S. verrucosus, S. celebensis, S. cebifrons,* and *S. barbatus*).

The results from the four methods were normalised based on ranked genes, considering only genes that fell outside 99% of the parameter distribution as significant departures from a strict neutral expectation for each method. To ensure a more rigorous selection of candidates for cold adaptation, we classified genes as positively selected only if at least three of the four methods supported them (see Additional file 1, Table S7).

Initially, positively selected sites were identified using a conservative approach, which involved calculating the largest allele frequency difference (*ΔdAF*_cold-warm_) between cold- and warm-region wild boar populations for both regulatory and exonic variants. We further validated the signal of recent positive selection using the Extended Haplotype Homozygosity (EHH) method applied to the focal variants [58], as implemented in rehh v2 [59]. The ancestral-derived relationships were inferred based on polarising sites from the outgroup *Sus* species. We estimated the local phylogeny using FastTree v2.1 [60], based on haplotype consensus sequences generated with SAMtools and BCFtools v1.15 [43]. Finally, we assessed potential convergent evolution by comparing our identified candidate genes with genes that have been reported to be under positive selection in cold regions for other species (human [3] and cattle [61]).

### Functional pathway enrichment analysis for genes supported by at least three methods and allele frequency distribution for focal variant(s)

The intricate nature of climatic adaptation involves the responsiveness of hundreds of genes, many of which are likely under positive selection [3, 62]. To better understand the overall patterns of biological pathways, we conducted a functional enrichment analysis. For this analysis, we included only genes that were supported by at least three different methods, utilising the Metascape database [63].

We examined the origin of the derived allele by analysing allele frequency distributions across different populations and species. The population distribution analysis was based on an extended dataset of 488 samples (Additional file 1, Tables S2 and S10). We defined the focal allele across species as fixed if the homozygous allele was present in all outgroup samples.

To validate the presence or absence of an orthologous mutation, we used Ensembl v105 (https://dec2021.archive.ensembl.org/index.html). This allowed us to confirm presence or absence based on a whole-genome alignment between *S. scrofa* (Suidae) and *Catagonus wagneri,* a species from the sister family Tayassuidae.

### The CUT&Tag and RNA-seq data collection and processing

To investigate whether the identified variants under selection have distinct functional effects in the cold-adapted population, we performed functional assays and RNA-seq analysis. The samples for Cleavage Under Targets and Tagmentation (CUT&Tag) and RNA-seq were collected from 180-day-old Min pigs, a local breed from Northeast China known for its cold adaptation [64]. We specifically chose 180-day-old pigs to control for age-related variability in gene expression, ensuring that the observed differences reflect genetic mechanisms related to cold adaptation rather than developmental effects.

Tissue samples were snap-frozen in liquid nitrogen immediately after collection to preserve RNA integrity and histone modifications. All sample collections were approved by the Ethics Committee of Huazhong Agricultural University (2022-0031). The CUT&Tag experiment followed the original CUT&Tag guidelines for tissues [65]. The H3K27ac histone modification antibody was employed for antibody enrichment in the CUT&Tag analysis of fat and diencephalon tissues from the Min pig [66].

The RNA-seq protocols for fat and diencephalon tissues were based on rRNA-depletion and strand-specific RNA-seq methods provided by Illumina (Illumina, San Diego, CA). Sequencing for both CUT&Tag and RNA-seq was performed using the Illumina NovaSeq6000 (PE150) platform.

The sequencing data from CUT&Tag were cleaned using Cutadapt to remove the adapter sequences [67]. The clean data were then mapped to the Sus scrofa 11.1 reference genome using Bowtie2 [68]. Peak calling was performed with MACS [69]. The vertebrate motifs referenced in JASPAR2020 were utilized to match the enhancer sequence of focal genes [70]. The RNA-seq data were processed in accordance with our previous study [71].

### The origin of candidate variants under positive selection

There are four candidate scenarios that could explain the evolutionary origin of the derived alleles under positive selection. In the first scenario, the two alleles emerged de novo in the cold-region population. In the second scenario, the low-frequency standing variant was transferred from warm-region populations to cold-region populations through gene flow. In the third scenario, the two alleles appeared de novo in domestic pigs within the last 10,000 years and were subsequently transferred to cold-region wild boar populations via gene flow. And in the fourth scenario, low-frequency standing variation existed at the time of divergence of the Northern populations and later increased in frequency due to selection or drift.

To differentiate between the first two scenarios, we performed gene flow analysis on the genomic region surrounding the focal variants. Specifically, we conducted the localised TreeMix analysis to determine the direction of gene flow [45]. Additionally, we confirmed potential gene flow by analysing the phylogenetic discordance between local background topologies, using a method similar to that of the previous study [17]. The third scenario would be valid if the allele frequencies of the positively selected derived alleles are significantly higher in domestic pigs compared to wild boar populations. The fourth scenario is challenging to assess in this study due to the lack of ancient Asian wild boar samples.

### Historical population demography for cold-region populations

To explore the demographic history of tropical Asian wild boar and Siberian wild boar, we conducted the PopSizeABC analysis [72], using the same samples as used for the selection scan. Historical demographic analysis can help us ascertain whether the signals of natural selection we identified are instead due to genetic drift. This is important because genetic drift, which can occur when the effective population size (Ne) is small, might lead to the random fixation of neutral variants [73].

Specifically, we began by summarising the folded allele frequency spectrum and the average zygotic linkage disequilibrium. Next, we simulated 400,000 datasets based on random population size histories. These pseudo-observed datasets (PODs) matched the sample size and covered 100 independent regions, each 2 Mb long. We independently estimated ancestral population sizes using the ABC method for each POD. Finally, we compared the estimated values to the true values, with a tolerance rate of 0.001.

## Results

### The phylogenetic origin of wild boar from cold and warm regions

**Fig. 1:**
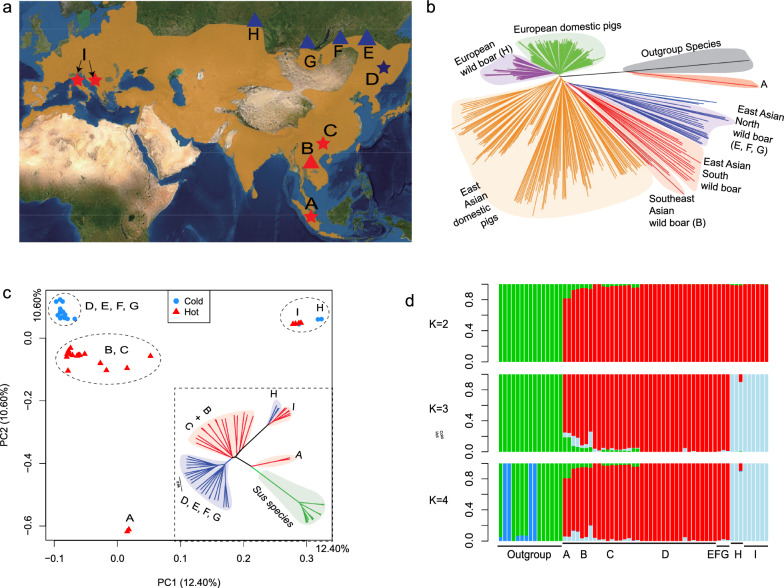
The population distribution, phylogeny, structure, and Admixture analysis of wild boar populations. **a** The wild boar population distribution (map retrieved from the IUCN red list: https://www.iucnredlist.org/species/41775/44141833). The red and blue colors indicate samples from warm and cold regions, respectively. Triangles and stars indicate populations with newly sequenced data and publicly available data, respectively. The population locations are Sumatra (A), Vietnam (B), South China (C), Northeast China and Korea (D), Zabaykalsky Krai (E), Buryat (F), Tyva (G), Novosibirsk (H), and Italy and Greece (I). **b** The phylogenetic tree for the extended dataset of 448 samples is based on IBS distances among samples. Background shadows highlight major clades, and brackets indicate newly sequenced samples. **c** The principal component analysis (PCA) and phylogenetic tree based on autosomal SNPs. In the phylogenetic tree, all major clade divisions were supported by 1000 bootstrap replicates (100%). **d** The admixture analysis (K = 2, 3, 4) for population ancestry. The green color indicates the outgroup *Sus* species (*S. verrucosus, S. celebensis, S. cebifrons,* and *S. barbatus*) from the islands of Southeast Asia. The red symbols show the Southeast and East Asian population ancestry in populations from A to G. The light blue shows the European ancestry in H and I

We obtained a total of 821 Gb of whole-genome data from 11 new samples: six wild boars from Siberia and five from Southeast Asia (Fig. 1a). After mapping the data to the pig genome reference (Sscrofa v11.1), the average sequencing depth was estimated to be 28.32x. We compiled two datasets for different analytical purposes, each with varying sample sizes: a core dataset comprising 63 genomes and an extended dataset encompassing 488 genomes (see Table 1, Additional file 1 Table S1 and Table S2).

Using a distance matrix based on IBS, along with the neighbor-joining method, we initially evaluated the phylogenetic relationships of all 488 samples (Fig. 1b). Our analysis revealed that the eastern Siberian samples (E, F, and G in Fig. 1a) clustered within the clade of wild boar populations from northern China, northeastern China, and northeastern Asia, including South Korea and Japan (D). In contrast, the western Siberian samples (H in Fig. 1a) clustered with the European wild boar, while the southeastern Asian wild boar showed close clustering with wild boar and indigenous breeds from southern China.

For the core dataset, we conducted IBS phylogenetic inference and performed PCA to validate population relationships (Fig. 1c). These analyses showed a similar population phylogeny to that obtained with the extended dataset. Specifically, our newly sequenced tropical population (B) clustered together with the temperate wild boar from southern China (C in Fig. 1a). The newly sequenced Siberian population was divided into two clusters (E, F, and G vs. H, Fig. 1c), which was consistent with the pattern observed in the extended dataset (Fig. 1b). These results demonstrated a greater divergence between populations from western (H) and eastern Siberia (E, F, and G). In contrast, the tropical population (B, Vietnam) showed a close relationship with the temperate Asian wild boar populations (southern China, C). Overall, these findings indicate a substantial genomic differentiation between the peripheral populations in cold regions and the source populations in tropical regions.

**Fig. 2:**
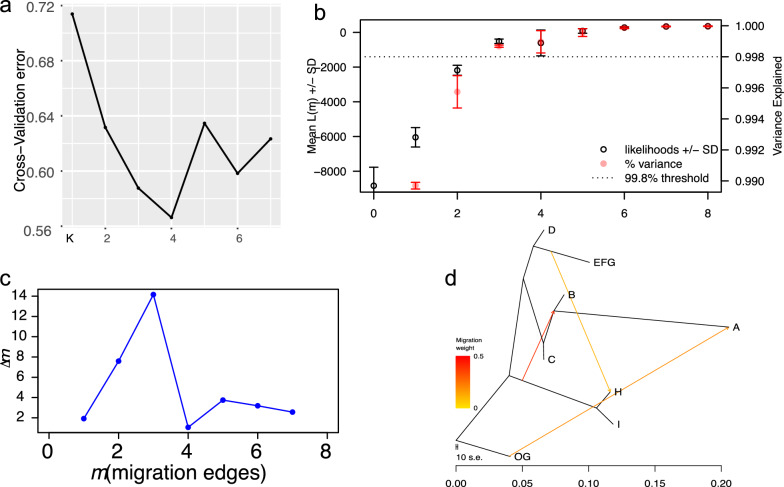
ADMIXTURE and gene flow analyses among major populations. **a** The cross-validation errors of the ADMIXTURE tool for inferring population ancestry and admixture. **b** The optimal number of migration events (m) for TreeMix based on the inference of OptM estimation. Over 99.8% of the variance was explained when m = 3. **c** The δm estimation supported the migration events of 3. **d** The direction of gene flow revealed by TreeMix, based on autosomal SNPs. The gene flow from eastern to western Siberia was detected (EFG to H). Three arrows show the directions from donor populations to recipient populations. Note: all the population codes are the same as in Fig. 1a

Further analysis on population structure was conducted using ADMIXTURE v1.3 [47] (Fig. 1d). A cross-validation approach identified the optimal number of distinct ancestries at K = 4 (Fig. 2a). The investigation of ancestral composition, considering ancestry counts ranging from two to four, revealed two primary clades that aligned with the established ancestries of Eurasian wild boar and domestic pigs, namely Asian and European lineages.

To examine gene flow between western and eastern Siberian populations, we utilised TreeMix (v1.12) [45] (Fig. 2). This analysis determined the optimal number of migration events (m) to be three, which accounted for over 99.8% of the variance in genetic relatedness among the populations (Fig. 2b, c). Notably, the inferred migration events indicated a predominant westward gene flow from eastern to western Siberian populations (Fig. 2d).

This division was further supported by PCA and phylogenetic assessments, which clarified the major population relationships (Figs. 1b and c). Interestingly, the population in western Siberia exhibited a 9.64% composition of Asian ancestry when analysed at K = 3 (Fig. 1d), suggesting limited gene flow from Asia into the western Siberian demographic.

### Genes under selective sweeps and their functional enrichment in thermogenic and adipose-related pathways

We used four complementary analytical approaches to identify candidate genes that exhibit signs of selective pressure in cold-region wild boar, specifically those native to Siberia, northern and northeastern China, and northeastern Asia (see Additional file, 1 Table S2–7). Our comprehensive analysis revealed that 1.54% of genes (313 out of 20,306, Ensembl v105) were consistently identified as being under selection by at least three of the four methods, reflecting conservative estimation. Furthermore, 0.45% of the identified genes (92 out of 20,306) were confirmed by all four analytical methods (see Additional file, 1 Table S7).

**Fig. 3:**
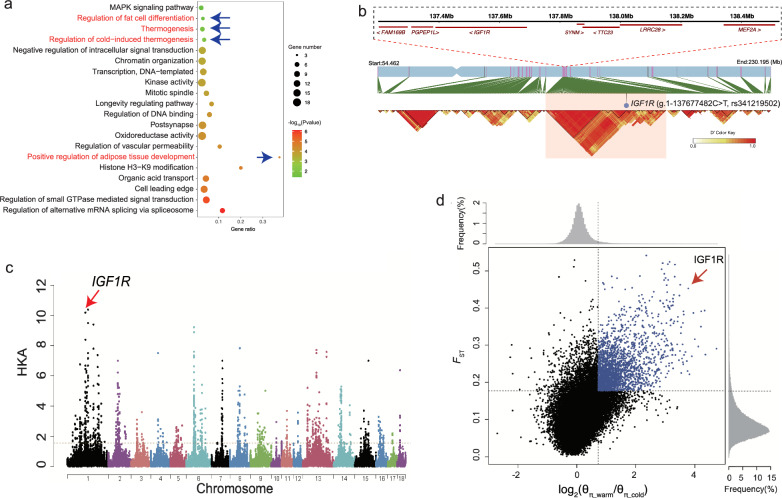
Functional enrichment analysis and selection signals for the candidate gene *IGF1R* underlying cold adaptation in the cold-region wild boar. **a** The enriched pathways were analyzed using the Metascape database. Only the significantly enriched pathways (*p* < 0.05) are listed (see Additional file 1 Table 8). **b** Haplotype blocks around *IGF1R* for the cold and warm region samples based on variants with allele frequencies higher in cold than in warm regions (*ΔdAF*_cold-warm_ > 0.5). **c** The plot of the HKA-like test. The vertical axis shows the decreased numbers of polymorphic sites relative to the counts of interspecies divergent sites. **d** The distribution of the Fixation index (*F*_*st*_) and nucleotide diversity ratio [log2(θ_π_warm_/θ_π_cold_)]

We analysed the functional enrichment of genes that were supported by at least three methods (Fig. 3a, Table 2, and Additional file 1, Table S8). This analysis revealed four pathways that may be related to cold resistance: the ‘thermogenesis’ pathway (*ADCY9*, *NDUFB6*, *PPARG*, PRKACB, *SMARCC1*, *TSC2*); the ‘regulation of cold-induced thermogenesis’ pathway (*ACADL*, *IGF1R*, *JAK2*, *NOVA1*, *NOVA2*); the ‘positive regulation of adipose tissue development’ pathway (*PPARG*, *NCOA2*, *SIRT1*); and the ‘fat cell differentiation’ pathway (*PPARG*, *TGFB1*, *SIRT1*, *WWTR1*) (*p* < 0.05, Fig. 3a). These findings suggest that specific pathways, particularly those regulating thermogenesis and fat cell differentiation, were crucial for the adaptation of cold-region wild boars to their harsh environmental conditions.

**Table 2:** Functional enrichment analysis of 305 genes identified to have been subject to selection for cold adaptation by at least three methods (see Additional File 1: Table S8 for details)

Category	Description	Symbols
GO BP	regulation of alternative mRNA splicing, via spliceosome	*MBNL1, NOVA1, NOVA2, TIA1, SMU1, CELF5, YTHDC1*
KEGG	Thermogenesis	*ADCY9, NDUFB6, PPARG, PRKACB, SMARCC1, TSC2*
GO BP	regulation of small GTPase mediated signal transduction	*DNM2, DOCK3, GPR4, ARHGAP35, ITGAV, MYO9B, RASA2, TSC2, LZTR1, ARHGEF12, DENND4C, ERBIN, RALGAPA1*
KEGG	MAPK signaling pathway	*CRKL, MECOM, IGF1R, PRKACB, RASA2, TGFB1*
GO BP	regulation of cold-induced thermogenesis	*ACADL, IGF1R, JAK2, NOVA1, NOVA2*
GO CC	cell leading edge	*CDK6, DNM2, GABRA5, ITGAV, MKLN1, MYO5A, SGCE, INPP5J, CTNNA3, DUOX2, DUOX1, TRPM7, MTMR14, DUOXA2*
GO BP	organic acid transport	*AQP9, GRM2, PPARG, SLC1A5, SLC1A6, ABCC5, SLC25A13, PLA2G3, SLCO1C1, SLC25A21, SFXN2*
GO BP	Histone H3-K9 modification	*MECOM, PRDM5, SIRT1, SETD5*
GO BP	positive regulation of adipose tissue development	*PPARG, NCOA2, SIRT1*
GO BP	regulation of vascular permeability	*GPR4, ARHGAP35, PDE3A, TGFB1, YES1*
GO MF	oxidoreductase activity	*ACADL, ALDH2, HMOX2, CYP4F3, NDUFB6, ALDH18A1, SORD, ALDH1A2, TXNRD2, CYP4F8, CYP2S1, DUOX2, PCYOX1, DUOX1, JMJD4, SELENOM, AIFM3, MSRB3*
GO CC	postsynapse	*AP2S1, CRKL, DCC, DNM2, GABRA5, GABRB3, GRIK2, GRM2, JAK2, MKLN1, TSC2, CAMK1, RNF10, STRN4, ERBIN, LHFPL4*
GO BP	regulation of DNA binding	*BCL3, CALM3, ERCC2, JAK2, PPARG, TGFB1, TNKS*
KEGG	Longevity regulating pathway	*ADCY9, IGF1R, PPARG, PRKACB, TSC2, SIRT1*
GO BP	regulation of fat cell differentiation	*PPARG, TGFB1, SIRT1, WWTR1*
GO CC	mitotic spindle	*LIMK2, MAP4, TNKS, MAPKBP1, TOGARAM1, TBL1XR1, TBCK, HEPACAM2*
GO MF	kinase activity	*AXL, CDK6, GALK2, IGF1R, JAK2, LIMK2, MATK, PI4KA, PRKACB, ALDH18A1, YES1, CAMK1, BRD4, PRKD2, STK39, TRPM7, TBCK*
GO BP	transcription, DNA-templated	*C5AR1, ERCC2, FOSB, MEF2A, DRG1, NFIC, PPARG, NCOA2, PPP1R13L, POLR1G, MKRN2, HIF3A*
GO BP	chromatin organization	*MECOM, FOXA3, JAK2, SMARCC1, BRPF1, PRDM5, RAD54L2, SIRT1, BRD4, CECR2, SETD5, METTL4, TBL1XR1, YTHDC1, ARID2*
GO BP	negative regulation of intracellular signal transduction	*MECOM, ARHGAP35, IGF1R, PDE3A, PDE4D, PPARG, RASA2, TSC2, LZTR1, MAPKBP1, SIRT1, MKRN2, CBLC, ERBIN*

By analysing genetic variants with higher allele frequencies in wild boar from cold region (Siberia, Korea, northern and northeastern China) compared to those from warmer regions (temperate and tropical Asia), we discovered a long haplotype block that exhibited strong linkage disequilibrium (LD, *ΔdAF*_*cold-warm*_ > 0.5, D’ > 0.9) among the variants of seven genes (*FAM169B*, *PGPEP1L*, *IGF1R*, *SYNM*, *TTC23*, *LRRC28*, and *MEF2A*). Notably, this region was the longest gene cluster we identified showing selective signals, spanning 1.3 Mb on chromosome 1 (137.2 Mb–138.5 Mb, Fig. 3b).

Among the linked genes, the Insulin-like Growth Factor 1 Receptor (*IGF1R*) received support from all four evaluation methods (see Additional file 1, Table S7). This suggests a reliable indication of positive selection that acted on this gene. Using the HKA-like test [51], we identified *IGF1R* as the most distinguished gene, demonstrating the highest reduction of polymorphism relative to divergence, which supports a significant deviation from neutral evolution (Fig. 3c).

At the population level, *IGF1R*, *ALDH1A2*, and *PGPEP1L* exhibited the highest inter-population divergences between wild boar from cold and warm regions among all protein-coding genes (*F*_*st*_ = 0.65, Fig. 3d and see Additional file 1, Table S3). Notably, among these three genes, *IGF1R* showed the greatest reduction in nucleotide diversity for wild boar from cold regions compared to those from warm regions (Fig. 3d), indicating strong positive selection on linked variants that are advantageous for cold adaptation.

### The potential convergent evolution for Siberian mammals

**Fig. 4:**
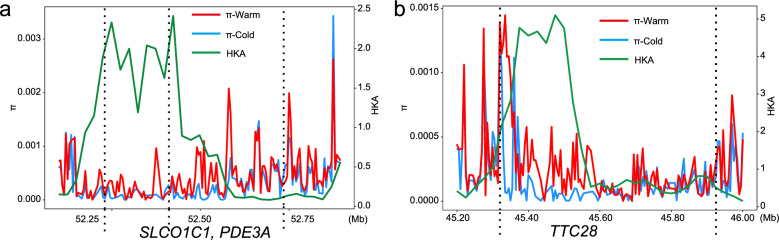
Selection signals for *SLCO1C1*, *PDE3A,* and *TTC28*. The warm-cold nucleotide diversity (π) comparison and HKA test (p < 0.01) for the neighboring genes *SLCO1C1* and *PDE3A* (**a**) and *TTC28* (**b**). Dotted lines indicate gene boundaries

The potential role of convergent evolution was examined by analysing genes under positive selection for cold adaptation in Siberian human populations. This analysis was conducted using the iHS and XP-EHH methods (top 1% windows) from a previous study [3] (see Additional file 1, Table S9). By concentrating on shared genes that were supported by four rigorous methods (see Additional file 1, Table S7), we identified three consensus genes, *SLCO1C1, PDE3A*, *and TTC28*, which suggests instances of convergent evolution. Additionally, we found positive selection signals by comparing nucleotide diversity between cold- and warm-regions populations and by conducting the HKA test. This analysis highlighted two neighboring genes, *SLCO1C1* and *PDE3A* (Fig. 4a), as well as another gene, *TTC28* (Fig. 4b).

### The most differentiated intronic and exonic variants were detected in *IGF1R *and* BRD4*, respectively

By analysing polymorphic variants across 92 candidate genes identified by four selection screening approaches (see Additional file 1, Table S7), we identified the variant with the greatest allele frequency difference (*ΔdAF*_cold-warm_) between cold- and warm-region wild boar populations for both regulatory and exonic variants. For regulatory variants, we identified the highest *ΔdAF*_cold-warm_ in an intronic variant of *IGF1R* (NC_010443.5:g.137677482C > T, c.94 + 12830G > A, intron 1, rs341219502), with *ΔdAF*_*cold-warm*_ = 0.896.

**Fig. 5:**
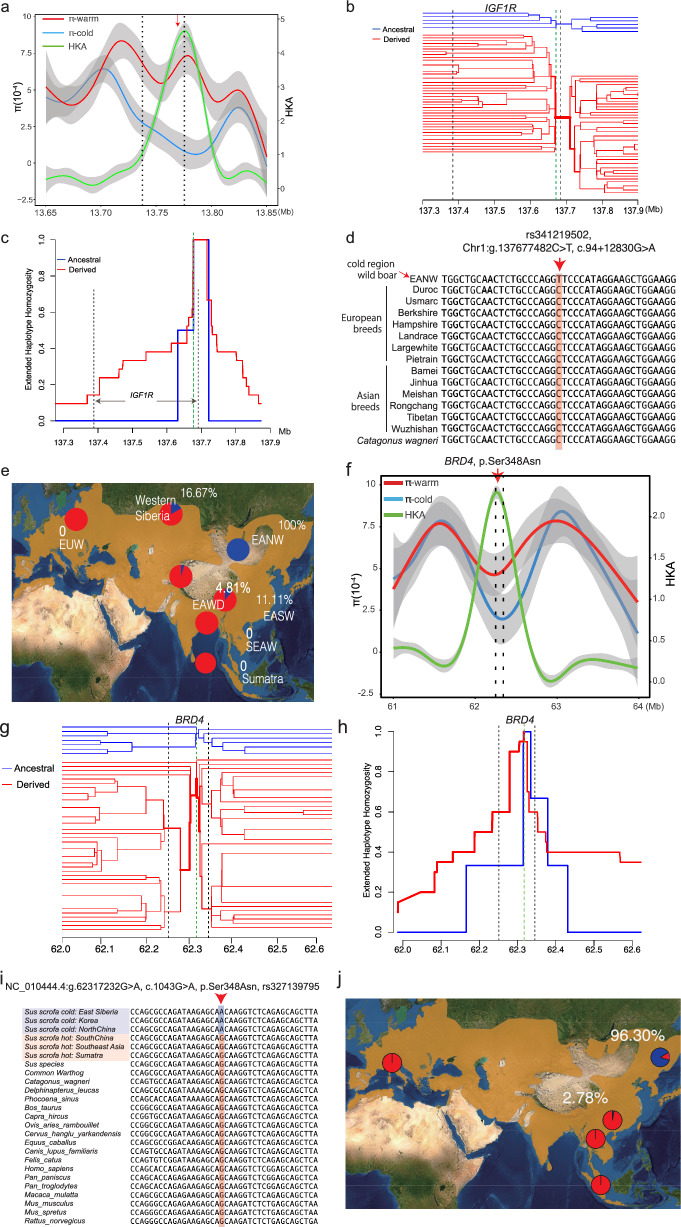
Site-level and haplotype-based selection signals, and allele frequency changes for *IGF1R* and *BRD4*. **a** The local signals of *IGF1R* selective sweep based on evidence of nucleotide diversity (π) for cold- and warm-region populations (left axis) and the polarized HKA test (right axis). The red arrow indicates the focal site of rs341219502 of *IGF1R*. **b** The EHH bifurcation diagram for haplotype density and breakdown around the site rs341219502 of *IGF1R*. Ancestral haplotypes are blue and derived ones are red. The line thickness is positively correlated to the density of haplotypes. **c** The EHH “hat” diagram for ancestral and derived haplotypes around *IGF1R* allele rs341219502 of *IGF1R*. **d** The population and evolutionary conservation for the ancestral state of rs341219502 (“C”) of *IGF1R* based on the whole-genome alignment. The first sequence represents the mutant sequence of cold-region wild boars. The next 13 sequences are retrieved from genomes of pig breeds in Ensembl (v105) and the last sequence shows *Catagonus wagneri* genome (Ensembl v105). **e** The allele frequency distribution for rs341219502 of *IGF1R* in multiple populations. **f** The local signals of *BRD4* based on evidence of nucleotide diversity (π) for cold- and warm-region populations (left axis) and the polarized HKA test (right axis). The red arrow indicates the focal site of rs327139795. **g** The EHH bifurcation diagram for haplotype density and breakdown around the site rs327139795 of *BRD4*. Ancestral haplotypes are blue and derived ones are red. The line thickness is positively correlated to the density of haplotypes. **h** The EHH “hat” diagram for ancestral and derived haplotypes around *BRD4* allele rs327139795. **i** The population and evolutionary conservation for the ancestral state of rs327139795 of *BRD4* based on the whole-genome alignment. **j** The allele frequency distribution for rs327139795 of *BRD4* in multiple populations

We validated selection on this variant using the method of extended haplotype homozygosity (EHH) [58, 74]. This showed a decay of haplotype homozygosity as the distance from the focal core allele increased (Fig. 5a). This evidence of positive selection was further supported by changes in nucleotide diversity (π) and the level of polymorphism relative to divergence between populations (Fig. 5a). The nucleotide diversity (π) near rs341219502 was significantly lower in cold-region wild boar populations compared to their warm-region counterparts (the chi-square test, *p* < 1.2 × 10^−4^). Similarly, we found a significant deficiency of derived polymorphic variants surrounding rs341219502 in cold-region wild boar relative to polarised divergent sites at the interspecific level (chi-square test, *p* < 2.2 × 10^−16^, red curve, Fig. 5b). The EHH decay was much more rapid for ancestral variant haplotypes (blue curve) than for derived variant haplotypes (red curve, Fig. 5c).

A cross-species orthologous alignment of Amniota vertebrates (Ensembl v105) revealed that the ancestral state ‘C’ was highly conserved in *Suidae* species, ranging from *Catagonus wagneri* to 13 pig breeds, suggesting that the'C'allele is likely the ancestral state (Fig. 5d). The allele frequency distribution indicated that this variant was absent in tropical Asian populations but fixed (100%) in northern Asian wild boar populations (Fig. 5e). All these findings support the recent selective sweep in the region surrounding rs341219502 of *IGF1R*.

For exonic variants, we identified those with disruptive or protein-altering effects. Based on the *ΔdAF*_cold-warm_ metric, we revealed a missense derived variant in the *BRD4* gene (NC_010444.4:g.62317232G > A, c.1043G > A, p.Ser348Asn, rs327139795) that exhibited the highest cold-warm differentiation (*ΔdAF*_cold-warm_ = 0.854) among all exonic variants analysed. We observed decreased nucleotide diversity in cold-region populations compared to warm-region populations, along with reduced polymorphism relative to divergence in cold-region populations (Fig. 5f). We also noted a delayed decay of derived haplotype homozygosity (Fig. 5g and h). These findings support the hypothesis that recent positive selection acted on this variant.

The nucleotide change from ‘G’ to ‘A’ results in an amino acid change from Ser to Asn in the second exon of *BRD4*. Multiple species alignment indicated that the ancestral state of the variant ‘G’ is highly conserved among mammals (Fig. 5i). We did not detect the derived allele ‘A’ in nine outgroup species, including African warthogs, pygmy hogs, and *Sus* species, which suggests that the derived allele ‘A’ likely emerged after the specification of *Sus* scrofa. We also found no evidence of the derived'A'allele in wild boar populations from Southeast Asia or Europe, indicating its origin in East Asian (Fig. 5i).

Within East Asian wild populations, the derived allele of rs327139795 was nearly fixed in cold-region populations (96.30%) but was a rare allele in warm-region populations (2.78%, Fig. 5j). Homozygotes for the derived allele “AA” were prevalent in cold-region populations (92.59%, 25/27) but completely absent in warm-region populations (0%, 0/18).

### Transcriptional changes in rs341219502 of *IGF1R* and post-translational changes in rs327139795 of *BRD4*

**Fig. 6:**
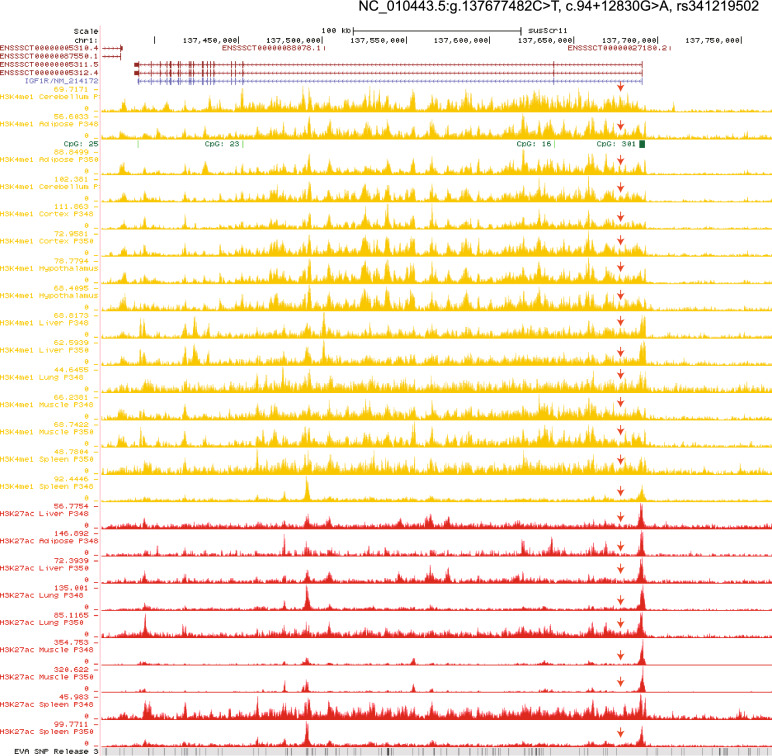
Mapping signals based on H3K4me1 and H3K27ac ChIP-seq data. The mapping signals based on H3K4me1 and H3K27ac ChIP-seq data retrieved from UCSC Genome Browser (http://genome.ucsc.edu/s/zhypan/susScr11_15_state_14_tissues_new). The red arrow shows the coordinate to the intronic variant rs341219502 of *IGF1R*

**Fig. 7:**
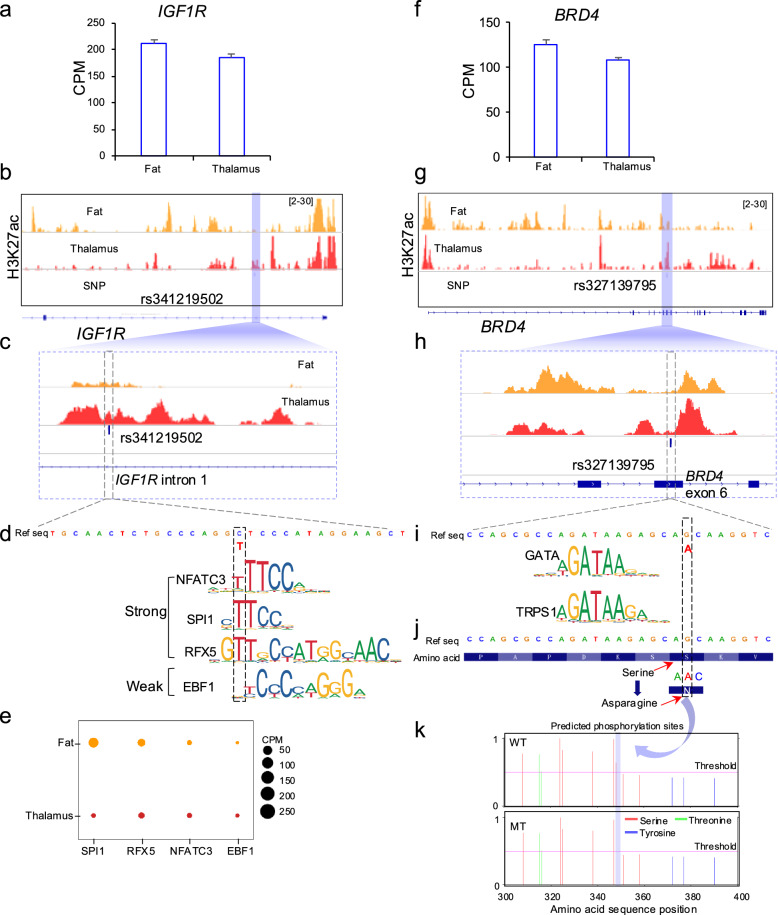
Regulatory enhancer mapping and mutational effects of rs341219502 in *IGF1R* and of rs327139795 in *BRD4*. **a** The RNA-seq expression of *IGF1R* in tissues of fat and diencephalon for the Min pig. **b** The H3K27ac intensity around the gene *IGF1R*. (c) The H3K27ac intensity around the rs341219502 in the *IGF1R* intron. **d** The predicted TF binding sites gained for rs341219502 (C > T) at *IGF1R* intron. **e** The RNA-seq expression profile of TFs in tissues of fat and diencephalon from the Min pig. Note: CPM, or Counts Per Million, is a gene expression normalization to make the expression levels comparable across different samples by accounting for sequencing depth and library size. **f** The expression of *BRD4* in fat and thalamus tissue of Min pig. **g** The H3K27ac intensity around the *BRD4* gene. **h** The H3K27ac intensity around the rs327139795 in *BRD4* exon. **i** The TF binding nearby the rs327139795. **j** The amino acid change of the rs327139795 in exon 6 of *BRD4*. **k** The absence of phosphorylation site in mutant type of rs327139795 in exon 6 of *BRD4*

Based on public data from H3K4me1 ChIP-seq of adipose and cerebellum tissue, we identified that the rs341219502 variant is located within the enhancer region of the first intron of *IGF1R* (Fig. 6). This suggests that this variant plays a significant role in regulating gene expression. To further explore this, we investigated the expression of *IGF1R* in the fat and diencephalon tissues of the Min pig, a local breed native to the cold region of northeastern China. We collected tissue samples from individuals carrying the mutant allele of *IGF1R*. Results from the RNA-seq expression analysis indicated that *IGF1R* is highly expressed in both the fat and diencephalon of adult Min pigs (Fig. 7a).

We conducted the CUT&Tag experiment [65] and identified the H3K27ac modification around the *IGF1R* gene (Fig. 7b). Specifically, we observed that signals related to the rs341219502 variant are within the enhancer region of the first intron of *IGF1R* (Fig. 7c). This finding confirms that this variant plays a role in regulating gene expression.

Furthermore, our prediction of the transcription factor (TF) motif showed that the derived allele ‘T’ introduces three novel TF binding sites, including the NFATC3, SPI1, and RFX5 (Fig. 7d). We then analysed the expression levels of these three TFs using RNA-seq and found that they are consistently expressed in adipose and diencephalon tissues (Fig. 7e). These results indicate that the rs341219502 derived allele ‘T’ acts as an enhancer mutation, potentially increasing the activity of the IGF1R enhancer by creating new binding sites for these transcription factors.

We also conducted an analysis of *BRD4*, a gene expressed in both adipose tissues and the diencephalon. We observed enrichment of the H3K27ac modification around *BRD4* and its exonic variant, rs327139795 (Fig. 7f–h). In contrast, transcription factor (TF) motif analyses did not identify any TF binding sites at the locus of the *BRD4* exonic variant rs327139795 (Fig. 7i).

Since this variant is located in the exon of *BRD4*, we further examined the amino acid changes associated with allele G and A (rs327139795). The results indicated that when the genomic sequence changes from G to A, the amino acid at this position (348aa) shifts from Serine to Asparagine (Fig. 7j). Serine is typically a site for phosphorylation and substituting it with Asparagine is predicted to eliminate this phosphorylation capability. To confirm this prediction, we performed a phosphorylation site analysis of both the wild-type *BRD4* and the rs327139795 variant using NetPhos 3.1 software, which employs neural network ensembles. The analysis verified the loss of the phosphorylation site at amino acid position 348 due to the substitution of Serine with Asparagine (Fig. 7k).

### Potential de novo origin and recent selective sweep of rs341219502 and rs327139795 in cold-region wild boar

**Fig. 8:**
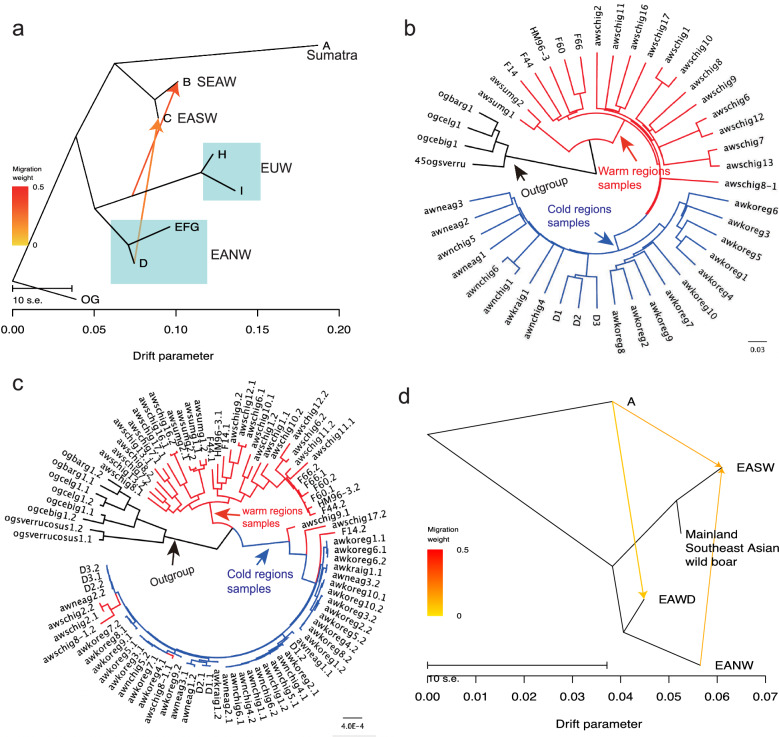
Derived allele frequency distribution and gene flow direction for rs341219502 and rs327139795. Description: **a** The localized gene flow around variant rs341219502 (600 Kb) revealed by TreeMix. Three arrows show the directions from donor populations to recipient populations. All population codes are the same as in Fig. 1a. **b** The background topology of chromosome 1 with a highlight on the two clades of North-region (cold) and South-region (warm) populations, represented by blue and red branches, respectively. **c** The local haplotype tree of 600 Kb around the rs341219502 inferred with the Maximum likelihood method of FastTree v2.1. The red haplotypes from the warm-region population were nested into the clade of the cold-region population. The “1” and “2” represent the two haplotypes for each sample. The black asterisks indicate major clade branching with 100% support values. The triangles represent wild boar samples from temperate or tropical regions that are nested within the clade of the cold region. The abbreviations of regions are: EUW, European wild boar; EAWD, East Asian western domestic pigs (Tibetan breed); SEAW, southeastern Asian wild boar; EASW, East Asian southern wild boar; EANW, East Asian northern wild boar (including populations from northern China, Korea, eastern Siberia, and northeastern China). **d** The localized TreeMix migration events (m = 3) for the region from 61.5 Mb to 63 Mb around the 811 selected variant rs327139795 of *BRD4*. The directions of gene flow are shown with arrows

The localised TreeMix analysis of the genomic regions upstream and downstream of the intronic variant rs341219502 in *IGF1R* (137.3 Mb–137.6 Mb) indicated a gene flow from cold-region populations to warm-region populations (Fig. 8a, EANW to EASW). Phylogenetic relationships confirmed this direction of gene flow (Fig. 8b). Specifically, within the background topology of chromosome 1, East Asian wild boar populations were divided into a warm clade and cold clade (Fig. 8b).

However, the local haplotype tree around rs341219502 revealed that five haplotypes from the warm clade had dispersed into the cold clade (Fig. 8c). This suggests that some warm-clade haplotypes may have been replaced by those from the cold clade, indicating a typical process of gene flow. Consequently, the presence of the derived ‘T’ allele in two warm-region wild boar samples likely resulted from southward gene flow from the cold-region populations.

For gene flow analysis of the exonic variant rs327139795, we noted a small number of genic variants (only 21 within the gene). Therefore, we expanded our analysis to encompass broader surrounding regions of the focal variant (61.5 to 63 Mb) and estimated local gene flow events using TreeMix [45]. The results supported a gene flow direction from cold- to warm-region wild boars (Fig. 8d). This observation suggests that the low frequency of allele ‘A’ in rs327139795 within the warm-region population was likely introduced into the warm-region population via gene flow from cold regions.

To evaluate the likelihood of the third scenario, we analysed the allele frequency distribution among domestic pigs (353 samples, see Additional file 1, Table S10). We did not find the derived ‘T’ allele of rs341219502 in either European or southern Chinese domestic pig populations. This allele was present as a rare variant in East Asian northern and western domestic pigs, with low frequencies (2.98 and 4.81%, respectively). Only 2.55% (9/353) of the northern Chinese domestic samples carried this derived allele, specifically in Min, Meishan, and Tibetan breeds. Furthermore, the majority of the domestic genotypes (8/9) that carried the derived allele were heterozygous, with only one Tibetan domestic pig being homozygous. In sharp contrast, in cold-region wild populations from Japan, Korea, Siberia, and northern China, 93.3% (28/30) of the genotypes carrying this allele were homozygous. For rs327139795, homozygotes for allele ‘A’ were also rare in both European and Chinese domestic populations (1.07 and 6.58%, respectively). Overall, among all domestic pigs, individuals homozygous for the variant were rare, comprising just 0.28% (1/353) of the population for rs341219502.

## Discussion

### Positively selected genes, functional enrichment, convergent evolution, and functional studies

A key challenge in biology is to identify the functional elements within the genome and understand their roles in adaptive processes. Natural selection can produce distinctive patterns in the genomic regions surrounding positively selected genes, which differ from those predicted under neutral evolution. Several key indicators, such as variations in genetic diversity, shifts in allele frequencies, and divergences between species, are essential tools for identifying genes under positive selection that are vital for complex adaptations [15, 17, 75–78].

For instance, research on the plateau wild boar has revealed genes with selective signals that are crucial for surviving harsh environmental conditions [78–81]. Additionally, the haplotype approach has identified a significant section of the X chromosome involved in climate adaptation in both wild and domestic pig populations from northern China and Europe [17]. These studies have greatly enhanced our understanding of the dynamics of natural selection.

Northern Asia, particularly the vast expanse of Siberia, is known for its extremely cold winters, which create significant challenges for endothermic mammals in maintaining their thermal balance. The severe low temperatures act as a strong selective force, especially for species like the wild boar, requiring adaptations that enhance thermoregulation in these frigid conditions. In addition to the cold, another major environmental challenge is the scarcity of food resources during the long winters [21]. Therefore, investigating the molecular adaptations that allow peripheral or derived populations of wild boar to survive and thrive in the cold Siberian climate and its surrounding regions is a topic of great scientific interest.

In this study, we utilised whole-genome sequencing and four selective sweep scan methodologies to identify candidate genes involved in cold adaptation. Our pathway analysis highlighted several key biological processes, particularly those related to regulation of fat cell differentiation, development of adipose tissue, thermogenesis, and cold-induced thermogenesis. These findings confirm the critical role of brown adipose tissue in facilitating thermogenic adaptation to cold conditions. This process triggers a range of responses to cold, including neural, vascular, and metabolic responses, as demonstrated in research conducted on humans, mice, insects, and polar mammals [82–85].

We also explored the hypothesis that endothermic mammalian species living in cold regions, such as Siberia, may share genes that have undergone convergent selection for cold resistance. Our analysis identified three positively selected genes *(SLCO1C1, PDE3A*, and *TTC28*) that have also been reported in indigenous Siberian human populations [3]. Notably, the relationship between low temperature and SNPs near *SLCO1C1 and PDE3A* has been also observed in Holstein cattle [61]. Consequently, it is likely that these genes are subject to recurrent convergent evolution in mammals due to their interconnected functions related to cold resistance [62, 86].

Two genes, *IGF1R* and *BRD4*, were supported by four methods and showed the strongest site-level selection signals. *IGF1R*, in particular, is intriguing due to extensive research on its functions relating to thermoregulation. Studies involving transgenic mice have demonstrated that *IGF1R* can lower core body temperature when subjected to the combined effects of cold stress and calorie restriction [87, 88]. Additionally, *IGF1R* may play a role in regulating body size [89–91].

Natural selection, driven by emerging selective forces, can lead to an increased frequency or fixation of derived alleles, as well as a decrease in the frequency or loss of ancestral alleles within a peripheral population, due to novel adaptation [19, 58]. The variants that cause changes in genes undergoing positive selection are expected to show significant differences in allele frequencies between peripheral and source populations [19].

Among the identified variants, an intronic variant with likely regulatory impact (rs341219502, c.94 + 12830G > A, reverse strand) in *IGF1R* and a missense variant (rs327139795, c.1043G > A, forward strand) in *BRD4* displayed the strongest differentiation between populations from cold and warm regions. Notably, *BRD4* was also among the genes supported by all four methods of selective sweep scans.

Fat and diencephalon are among the tissues that contribute to an animal's ability to withstand cold temperatures [92, 93]. Our CUT&Tag experiment on these tissues indicates that the derived allele of rs341219502 is located in the enhancer region of *IGF1R*. We validated the enhancer signals of this variant in both fat and diencephalon tissues of the Min pig, a local breed adapted to the cold environment of northeastern China. The transition of the allele from ‘C’ to ‘T’ could create novel binding sites for TFs, including NFATC3, SPI1 and RFX5, at the enhancer region.

Interestingly, previous studies have shown that NFATC3 is essential for cardiac development and mitochondrial function [94]. Additionally, NFATC3 can enhance insulin sensitivity, influencing gene expression that affects the development and adaptation of various mammalian cell types, including adipocytes and neurons [95]. SPI1 (PU.1) has the ability to inhibit adipocyte differentiation [96, 97], while RFX5 is involved in regulating resistance to nutrient stress [98].

Moreover, the ‘A’ allele of rs327139795, which is located within the exon of *BRD4,* leads to a change from Serine to Asparagine. This alteration, based on the inherent characteristics of amino acids, could impact phosphorylation sites. As a result, this change may affect the interaction between the mutated Asparagine and the surrounding amino acids, potentially altering protein function.

Future research that integrates single-cell multi-omics and spatial transcriptomics may provide a clearer understanding of the functional mechanisms behind these variants, offering valuable insights into evolutionary adaptation and its practical applications in agriculture and conservation genetics.

### Evolutionary origin of the candidate variants under positive selection for cold resistance

The two mutations, ‘T’ of rs341219502 in *IGF1R* and ‘A’ of rs327139795 in *BRD4*, are absent in all the outgroup species from the Suidae and Tayassuidae families. This observation supports the idea that these variants are of recent origin rather than being ancient. At the population level, these alleles were fixed only in wild boar from cold regions, while they were rare or absent in wild boars from warmer regions, which are closer to the origin of *S. scrofa* (Southeast Asia). This strongly suggests that these mutations likely originated de novo in the wild populations of cold regions.

Although these two alleles were detected in a small fraction of the wild boar population in southern Chinese and in Tibetan domestic pigs, it is highly probable that they were introduced from northern populations through gene flow. This conclusion is based on evidence from local TreeMix signals (or phylogeny misplacement), very low allele frequencies, and an extremely high heterozygosity rate among individuals carrying these alleles in those population groups.

Our analysis of allele frequencies for candidate variants under positive selection suggests that these two derived alleles likely did not originate from domestic pigs. The patterns observed in the origin and emergence of rs341219502 and rs327139795 align with the most straightforward interpretation that these alleles originated de novo origin in wild boar populations. The alternative hypothesis—that these variants stem from ancestral polymorphism— is also less convincing, as no such variants have been identified in Southeast Asian and European wild boars or in outgroup species within the Suidae family.

Considering that the divergence time between Northern and Southern Chinese wild boars dates back 25,000 to 50,000 years [99, 100], it is likely that these two variants are less than 50,000 years old. After their de novo emergence, natural selection probably facilitated their fixation in wild populations located in colder regions. However, we cannot completely rule out the possibility that these variants originated from low-frequency standing variants that were present at the time of divergence of Northern Asian populations, which then increased to near fixation. Future extensive sampling of wild boar populations in colder regions could help clarify this question.

**Fig. 9:**
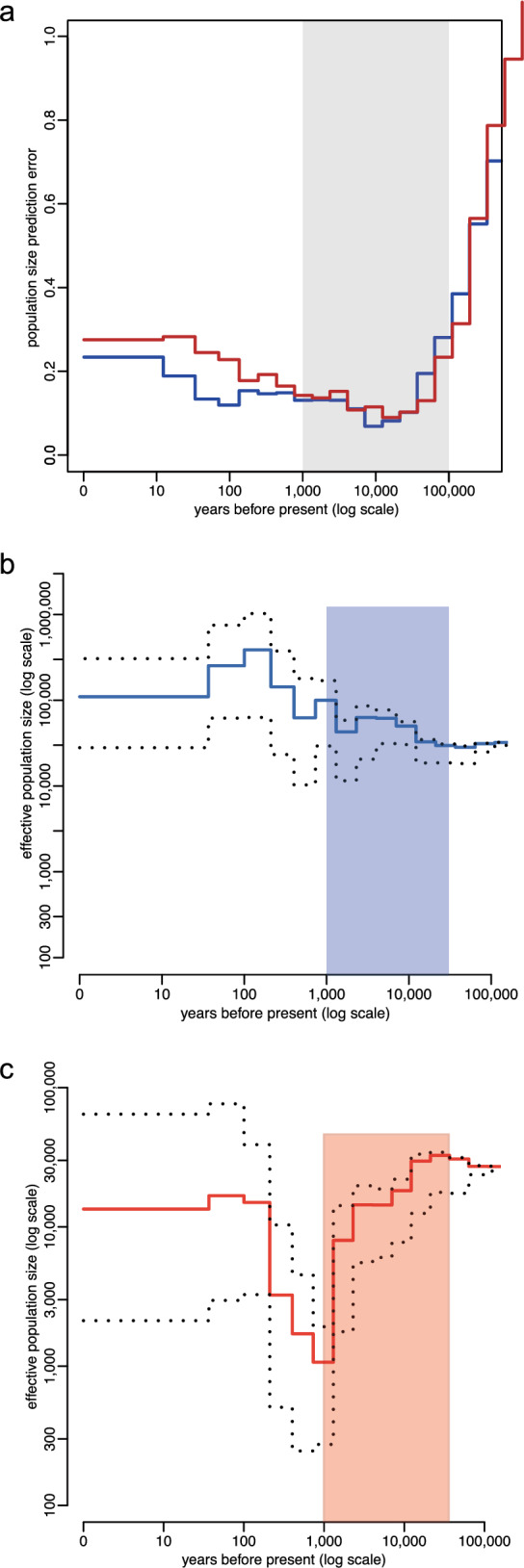
Historical effective population sizes inferred with the approximate Bayesian computation analysis. **a** The time range from 50,000 to 1000 years ago has the lowest errors (< 20%, scaled) under the tolerance rate of 0.001. The red and blue lines show the warm- and cold-region populations, respectively. **b** The historical demography of cold-region wild boar populations. **c** The historical demography of warm-region wild boar populations. The dotted lines indicate the 5% and 95% quantiles of the posterior distribution. The red and blue frames show the time range from 50,000 to 1000 years ago

Another open question is whether the selected alleles in cold region populations were near-fixed due to positive Darwinian selection or genetic drift. The strong signals of positive selection detected at these two sites (rs341219502 and rs327139795) support the first hypothesis. However, rapid genetic drift can sometimes produce patterns similar to those caused by positive selection [101]. Therefore, it is essential to further investigate the drift hypothesis. Since rapid genetic drift would necessitate a significant reduction in the historical effective population size, we formally evaluated this possibility using PopSizeABC, a simulation-based method for inferring demographic history under the framework of approximate Bayesian computation (ABC). We analysed the ancestral dynamics of effective population size for both warm- and cold-region wild populations of Asian origin. Based on population-level diploid genomes for warm- and cold-region populations, we identified distinct trends in demographic changes over the last 100,000 to 1000 years ago (Fig. 9).

Numerous studies have found that the divergence time between Northern and Southern Chinese wild boar ranges from 25,000 to 50,000 years ago [99, 100]. This indicates that wild boars may have arrived in northern China prior to this period, with the specified range representing the upper and lower limits of the divergence time between the two populations. Using an approximate Bayesian computation framework, which accommodates more samples than other tools (PSMC [102] and MSMC [103]) and is robust against sequencing errors and complex population dynamics [72], we reconstructed demographic history for both warm- and cold-region samples. This analysis suggested that the emergence of these variants is unlikely to be due to random effects (such as genetic drift). The prediction errors from the PopSizeABC inference were found to be within the acceptable range [72] (Fig. 9a).

PopSizeABC indicated a consistently increasing trend in historical population size for cold-region wild boar populations during the period of ~ 25,000 to 50,000 years ago (Fig. 9b). In contrast, the population sizes of warm-region wild boar populations sharply declined during this timeframe (Fig. 9c). Therefore, the increasing trend in the historical population size of cold-region wild boars does not support the hypothesis of rapid genetic drift.

Our findings lay the groundwork for enhancing cold-adapted livestock breeds through marker-assisted selection, utilizing both computational biology and experimental validation. Future studies involving larger sample sizes of wild boar or other free-ranging species from cold regions may reveal additional positively selected variants associated with cold resistance. These insights can be used to improve the economic performance of local domestic breeds while helping farmers reduce energy costs. For instance, more precise techniques like CRISPR-based genome editing could enhance thermoregulation and metabolic traits. Furthermore, advancements in AI-driven genomic prediction models could expedite the identification of adaptive variants across different populations, thereby informing data-driven breeding programs and simulations of gene-environment interactions in response to changing climatic conditions.

Similar methodologies and principles could be employed to explore various scenarios of climatic adaptation, including heat stress, cold tolerance, and high-altitude adaptation, yielding insights into the genetic and physiological mechanisms underlying environmental resilience.

## Conclusions

The wild boar has been remarkably successful in colonising Eurasia, including a rapid expansion from tropical Asia into a variety of climates. This expansion includes their movement into the extreme cold of arctic Siberia less than a million years ago. By employing whole-genome sequencing and various methods of summary statistics—such as analysing the allele frequency spectrum (*F*_*st*_ and the ratio of θ_π_), haplotype, and species divergence—we identified genes undergoing selective sweeps in populations from cold regions.

These genes were found to enhance metabolic pathways critical for cold resistance, which include thermogenesis, fat cell development, and the regulation of adipose tissue. The most significant selection signal was detected in a 1.3 Mb region on chromosome 1, characterised by linkage disequilibrium surrounding the *IGF1R* gene. At the variant level, a regulatory variant within *IGF1R* and a missense variant within *BRD4* showed the most pronounced differences in allele frequency between the warm- and cold-region populations. Analysis of allele frequency distributions suggested that these variants likely originated de novo within cold-region wild populations. Demographic reconstructions indicated that genetic drift is unlikely to have contributed to the emergence of these variants.

Given the known roles of *BRD4* and *IGF1R* in regulating bioenergy homeostasis and body temperature [87, 104, 105], our finds illuminate the molecular adaptations of wild boar populations cold climates in Siberia and its surrounding regions. Because cold stress is a leading cause of neonatal piglet mortality [105], our study could inform breeding programs aimed at enhancing piglet cold tolerance.

## Supplementary Information

Additional file 1: Table S1: Simplified core dataset . Table S2: Larger dataset . Table S3: Selective sweep method 1 based on diversity ratio and fixation index . Only the top 5% regions are shown. Table S4: Selective sweep method 2 based on diversity ratio and XP-CLR. Only the top 5% regions are shown.  Table S5: Selective sweep method 3 based on iHH12 based on top 1% SNPs. Table S6: Selective sweep method 4 based on HKA test based on top 1% SNPs. Table S7: Genes shared by three to four methods. Table S8: Gene functional enrichment for 305 genes shared by at least three methods. Table S9: Human Siberian populations selective signals based the report of Cardona et al. [3]. Table S10: Genotyping and group assignment for an intron variant in *IGF1R*  and an exonic variant in *BRD4* 

## Data Availability

The whole-genome sequence data can be accessed through NCBI BioProject code PRJNA859556.
